# Ratings of perceived exertion from a submaximal 20-m shuttle run test predict peak oxygen uptake in children and the test feels better

**DOI:** 10.1007/s00421-022-05047-6

**Published:** 2022-10-03

**Authors:** Daiki Kasai, Margarita D. Tsiros, Roger Eston, Gaynor Parfitt

**Affiliations:** 1 UniSA Allied Health and Human Performance, Alliance for Research in Exercise, Nutrition and Activity (ARENA), University of South Australia, City East Campus, Cnr of North Terrace & Frome Rd, Adelaide, SA 5001 Australia; 2 UniSA Allied Health and Human Performance, Innovation, Implementation and Clinical Translation in Health (IIMPACT), University of South Australia, Adelaide, SA 5001 Australia

**Keywords:** RPE, Oxygen uptake, Cardiorespiratory fitness, 20mSRT, Affect

## Abstract

**Purpose:**

To determine the validity and test–retest reliability of using ratings of perceived exertion (RPE) elicited during a submaximal 20-m Shuttle Run Test (20mSRT) to predict VO_2peak_ in children and investigate acute affective responses.

**Methods:**

Twenty-five children (14 boys; age, 12.8 ± 0.7 years; height, 162.0 ± 9.3 cm; mass, 49.9 ± 7.7 kg) completed four exercise tests (GXT, 2 submaximal 20mSRT, maximal 20mSRT). The Eston–Parfitt RPE scale was used, and affect was measured with the Feeling Scale. Submaximal 20mSRT were terminated upon participants reporting RPE7. The speed-RPE relationship from the submaximal 20mSRTs was extrapolated to RPE9 and 10 to predict peak speed and then used to estimate VO_2peak_.

**Results:**

Repeated measures ANOVA to examine the validity of using submaximal RPE to predict VO_2peak_ resulted in a Gender main effect (boys = 46.7 ± 5.1 mL kg^−1^ min^−1^; girls = 42.0 ± 5.1 mL kg^−1^ min^−1^) and Method main effect (*p* < 0.01). There were significant differences between measured and estimated VO_2peak_ from the maximal 20mSRT, but not between measured and estimated VO_2peak_ at RPE9 and RPE10. Intraclass correlation analysis revealed excellent reliability (~ 0.9) between the two submaximal 20mSRTs. Significant differences (*p* < 0.05) in end-test affect were reported between submaximal and maximal trials in girls, but not in boys, with girls feeling less negative at the end of the submaximal trials.

**Conclusions:**

The results of this study provide evidence that RPE reported during a submaximal 20mSRT can be used to predict VO_2peak_ accurately and reliably. In this study, the submaximal 20mSRT ending at RPE7, provided better predictions of VO_2peak_ while minimising aversive end-point affect, especially in girls.

## Introduction

Physical activity is associated with improvements in health-related fitness such as; cardiorespiratory fitness and plays a significant role in reducing the risk of many non-communicable diseases (Bull et al. 2020). Due to growing concern over physical activity levels and health-related fitness of children, standardised fitness testing has been introduced into physical education curriculums internationally (Cale et al. 2014). Health-related fitness, specifically cardiorespiratory fitness, is a strong predictor of future health outcomes (Kodama et al. 2009).

Cardiorespiratory fitness refers to an individual’s ability to perform exercise involving large muscle groups at a moderate to high intensity for an extended period of time (Tomkinson and Olds 2008). In children, peak oxygen uptake (VO_2peak_) is typically used instead of VO_2max_ as the criterion measure of cardiorespiratory fitness as children often do not exhibit a plateau in VO_2_ (Tomkinson and Olds 2008). Laboratory-based maximal graded exercise testing (GXT) with direct online gas analysis is considered the ‘gold-standard’ for assessing VO_2peak_. However, due to limitations such as; the need for expensive equipment, testing time and tester expertise, numerous field-based fitness tests have been developed to predict VO_2peak_ from submaximal or maximal bouts of exercise (Tomkinson and Olds 2008).

Field-based tests are a practical alternative to the GXT as they do not require an expensive laboratory-based setup and rely on measures, such as heart rate (HR) and/or distance/speed to predict VO_2peak_ through standardised equations, such as the American College of Sports Medicine’s (ACSM) metabolic equations (Tomkinson and Olds 2008; ACSM 2007). The 20-m shuttle-run test (20mSRT) is arguably the most popular field-based test and has previously been used in over 50 countries (Lang et al. 2018). In the 20mSRT, participants run repeatedly between two parallel lines 20-m apart in time with an audio signal that gets progressively faster until they reach volitional exhaustion. Currently, there are numerous equations designed to predict VO_2peak_ based on performance in the 20mSRT (Lang et al. 2018). However, all vary in validity and provide differences in estimates of VO_2peak_ (Lang et al. 2018). Despite the popularity and advantages, the 20mSRT involves maximal effort by the participant and, therefore, may induce acute negative affective responses.

‘Affect’ is a term that can be used to describe the subjective experience of any state that is ‘valenced’ (positive/pleasure or negative/displeasure) (Ekkekakis and Petruzzello 2002). The relationship between affective responses and exercise intensity is intricate (Ekkekakis 2003). The ‘dual-mode’ theory (Ekkekakis 2003) suggests that affective responses during exercise are the product of the interplay between cognitive processes (e.g., personality, attributions, goal orientations and self-efficacy) and interoceptive cues (e.g., signals from various sensory receptors) that occur at various exercise intensities. At moderate intensities (below Ventilatory Threshold [VT]), affective responses are positive with low interindividual variance (Ekkekakis 2003). Affective responses during exercise between VT and Maximal Lactate Steady State (MLSS) are subject to high interindividual differences due to how an individual may over-ride and interpret afferent interoceptive cues. However, as exercise intensity increases and exceeds MLSS, affective responses are uniformly negative due to the domination of interoceptive cues over cognitive processes to avoid physiological harm (Ekkekakis 2003). Acute affective responses to exercise are important to consider as it has been shown to be a strong predictor of future exercise participation. A one-unit increase in positive affective response during exercise was associated with ~ 30 min of moderate-to-vigorous physical activity (MVPA) per week in children (Schneider et al. 2009). The relationship between exercise intensities beyond MLSS and negative affect is, therefore, important to consider as children may be exposed to these intensities routinely through standardised fitness testing.

Whilst ‘how we feel’ during exercise is important to consider for future PA behaviours, our inherent ability to perceive how hard we are working during an exercise task can be used to predict exercise capacity. The use of the Rating of Perceived Exertion (RPE) has been found to be a valid method to predict VO_2peak_ (Eston and Parfitt 2018). The utility of RPE to predict VO_2peak_ is founded on its strong linear relationship with objective markers of exercise intensity (e.g., VO_2_, heart rate [HR], work rate) during aerobic exercise. This strong inherent association allows the relationship between RPE and its corresponding VO_2_ to be extrapolated to a perceptual end-point. The validity of predicting VO_2max/peak_ from submaximal RPE has been explored extensively in adults during laboratory-based exercise tests (Coquart et al. 2014) and during a simulated 20mSRT (Davies et al. 2008).

As RPE is moderated by psychological factors (e.g., cognition, memory, prior experience and task comprehension), child-specific scales have been developed to assess RPE in children (Eston and Parfitt 2018). Child-specific scales employ verbal descriptors and numerical ranges that are more familiar to children. However, research exploring the application of RPE to predict VO_2peak_ in children is limited. Lambrick et al. (2016) provided evidence to support the validity of predicting VO_2peak_ using submaximal RPE values during a single GXT in children. However, no studies to our knowledge have explored the validity and reliability of using submaximal RPE elicited during a field-based exercise test (i.e., 20mSRT) to predict VO_2peak_ in children. As exercise significantly beyond VT is likely to produce negative affective responses, a submaximal field-based test may be of benefit by being less affectually aversive while providing accurate predictions in VO_2peak_. Therefore, the objective of the study was to determine the validity and test–re-test reliability of using RPE elicited during a submaximal 20mSRT to predict VO_2peak_ in children and to assess affective responses. It was hypothesised that: speed obtained via individual RPE from a submaximal 20mSRT could be used to accurately and reliably predict VO_2peak_. It was also hypothesised that participants would report less negative affective responses during the submaximal 20mSRT in comparison to the maximal GXT and 20mSRT.

## Methods

### Participants

Based on a two-factor [Gender(2)*Method(4)] repeated measures ANOVA to test the hypothesis, for a medium effect size, with power of 80% and alpha at 0.05, the total sample size required was 24 (Faul et al. 2007). To account for attrition, 32 apparently healthy children aged 12–14 years with no known pre-existing injuries or conditions were recruited. Children with physical, neurological or intellectual disability; those with acute injuries or certain medical conditions (e.g., severe asthma) were excluded from the study. The study received ethics approval from the University of South Australia Human Research Ethics Committee (Ethics No. 201495) and conducted in accordance with the ethical standards laid down in the 1964 Declaration of Helsinki. Written informed consent was obtained from the parent/legal guardian and assent from the participant prior to testing.

### Assessment tools

#### Eston–Parfitt RPE scale

The Eston–Parfitt (E–P) 0–10 pictorial scale was used to assess perceived exertion. The scale depicts a character at various levels of exertion along a concave slope that progressively increases in gradient. The E–P scale contains verbal anchors from “very, very easy” (0), “very easy” (2), “easy” (3), “starting to get hard” (4), “very hard” (7) to “so hard I am going to stop” (10). The E–P scale has good reliability (ICC = 0.71–0.76) (Eston and Parfitt 2007) and has been validated for quantifying overall perceived exertion in children during treadmill exercise (*R*^2^ = 0.96) (Lambrick et al. 2011). The E–P scale has also previously been used to predict VO_2peak_ in children during a GXT (Lambrick et al. 2016).

#### Feeling scale

Affective valence was recorded during the four exercise tests using the feeling scale (FS) (Hardy and Rejeski 1989). Participants reported how they currently felt on a 11-point numerical scale ranging from − 5 to + 5, with verbal anchors of “very good” (+ 5), “good” (+ 3), “fairly good” (+ 1), “neutral” (0), “fairly bad” (− 1), “bad” (− 3), and “very bad” (− 5). The FS has been successfully applied in studies with adolescents (Schneider et al. 2009).

Participants were familiarised and instructed on how to employ both the E–P scale and the FS scale prior to commencing each testing session.

### Procedures

Participants completed four continuous incremental exercise tests in a non-randomised order. These comprised one maximal laboratory-based test and three field-based 20mSRT tests; two of which were submaximal. Each test was separated by ≥ 48 h (ACSM 2014). Prior to testing, participants were familiarised with the equipment and exercise protocols.

#### Laboratory-based assessment

Participants first performed a single GXT to volitional exhaustion on a treadmill (Trakmaster TMX425CP; FullVision Inc., Kansas, USA) in a laboratory maintained within 18–20 °C to assess VO_2peak_. Height and mass were measured using a stadiometer (SECA 213, Ecomed, NSW, Australia) and scales (BC-148, Tanita, Tokyo, Japan). The GXT commenced at 4.0 km h^−1^ and increased by 1.0 km h^−1^ every minute up to 8.0 km h^−1^, then the speed was increased by 0.5 km h^−1^ every minute. Treadmill gradient was fixed at 1% throughout testing to replicate oxygen costs associated with overground running (Jones and Doust 1996). Breath-by-breath respiratory gas was analysed via an online respiratory gas analyser (Metalyzer 3B; Cortex Biophysik GmbH, Leipzig, Germany) with a paediatric face mask (Hans Rudolph Inc., Kansas, USA). Volume and gas calibration were performed prior to each session as per manufacturer guidelines. HR was measured using a wireless chest-strap telemetry system (Polar Electro Oy, Kempele, Finland). Participants reported their RPE and affect prior to commencing the GXT and during the final 15 s of each minute. Testing was terminated when the participants were no longer able to maintain the required exercise intensity despite verbal encouragement.

#### Field-based assessment

Following the completion of the GXT, participants performed a modified 20mSRT protocol three times; two submaximal tests and one maximal test to volitional exhaustion. In the original 20mSRT protocol by Léger et al. (1984), the test begins at 8.5 km h^−1^ and increases by 0.5 km h^−1^ every minute. However, the starting speed has previously been reported as being too fast for a paediatric population (Tomkinson and Olds 2008). Therefore, the modified 20mSRT started at 4 km h^−1^ and increased by 1 km h^−1^ every minute up to 8 km h^−1^, whereafter the speed was incremented by 0.5 km h^−1^ every minute. Participants reported their RPE and affect prior to commencing the trial and during the last level of each stage prior to the speed increments by pointing to each scale. During the two submaximal 20mSRTs, the participants were informed that the test would stop after they report an RPE value of 7. RPE7 was used as the termination point as it corresponds to a high exercise intensity (Lambrick et al. 2016). In their study, RPE7 on the E–P Scale corresponded to 90% VO_2peak_ during treadmill running for both boys and girls aged 9–10 years.

Following completion of the two-submaximal 20mSRTs, the participants completed a maximal version of the modified 20mSRT. In this test, the procedures from the submaximal 20mSRT were replicated. However, rather than the test finishing when the participants reported RPE7, the test continued with verbal encouragement until participants reached volitional exhaustion.

### Data analysis

#### Descriptive data

To examine gender differences on descriptive data (age, height, mass, BMI, VO_2peak_, HR_max_ and RER), independent samples t-tests were conducted using SPSS (ver.25, IBM Analytics, USA). The VT was determined from the GXT data using the three-method procedure proposed by Gaskill et al. (2001). Breath-by-breath data were collapsed and averaged into 10 s intervals to allow clearer identification of the break point.

#### Validity of prediction equations to estimate VO_2peak_

The validity of various prediction equations to estimate VO_2peak_ were initially assessed. Mean measured VO_2peak_ from the GXT and predicted VO_2peak_ from the maximal 20mSRT using five different equations (Leger et al. 1988; ACSM 2007; Barnett et al. 1993; Matsuzaka et al. 2004; Mahar et al. 2006) were analysed via a one-way repeated measures ANOVA using SPSS. The most accurate equation to predict VO_2peak_ was used for subsequent analyses.

$${\rm ACSM}\, (2007): {{\rm VO}}_{2}=\left(0.2\times {S}_{1}\right)+\left(0.9\times {S}_{1}\times G\right)+3.5$$

$${\rm Barnett\, et\, al.}\, (1993): {{\rm VO}}_{{\rm 2peak}}=25.8-6.6\times {G}_{1}-0.2\times W+3.2\times {S}_{2}$$

$${\rm Leger\,et\,al.}\,(1988): {{\rm VO}}_{{\rm 2peak}}=31.025+3.238\times {S}_{2}-3.248\times A+0.1536\times {S}_{2}\times A$$

$${\rm Mahar\,et\,al.}\, (2006): {{\rm VO}}_{{\rm 2peak}}=47.438+L\times 0.242+{G}_{2}\times 5.134-W\times 0.197$$

$${\rm Matsuzaka\,et\,al.}\, (2004): {{\rm VO}}_{{\rm 2peak}}=25.9-2.21\times {G}_{1}-0.449\times A-0.831\times {\rm BMI}+4.12\times {S}_{2}$$

$${S}_{1}: {\rm speed}\, \left(m \, {min}^{-1}\right);\,G: {\rm fractional}\,{\rm grade}; \; W{:} \; {\rm body}\,{\rm mass}\,\left(kg\right);\; {S}_{2}{:} {\rm running}\,{\rm speed}\,\left(km \, {h}^{-1}\right)\,{\rm at\,the\,last\,completed\,stage;\,A:\,participan{t}}^{{\prime}}s\,{\rm age\,at\, last birthday}; {G}_{1}: {\rm gender}\, \left({\rm male}=0, {\rm female}=1\right); {\rm BMI}:\,{\rm body\,mass\,index}\, \left(kg \, {m}^{-2}\right);\; L{:} {\rm number\,of\,laps\,completed}; \; {G}_{2}{:} {\rm gender}\,(m=1,f=0)$$

#### Prediction of VO_2peak_ from submaximal 20mSRT RPE

**Fig. 1:**
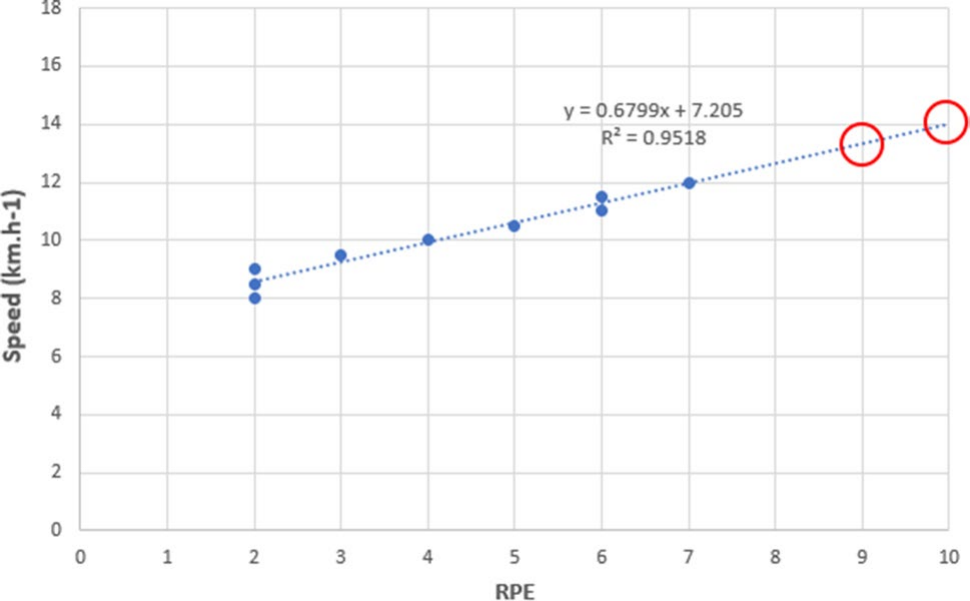
Example of a typical line of best fit for one of the study participants (boy, mass = 49.2 kg) from the first submaximal 20mSRT. The graph shows the strong linear relationship between exercise intensity (i.e., speed) and RPE. The line of best fit had an *R*^2^ of 0.95. Predicted maximum speed according to the linear model equation at RPE 9 and 10 were 13.3 km h^−1^ and 14 km h^−1^, respectively. The actual maximum speed achieved in the maximal SRT for this boy was 13.5 km h^−1^. Maximal treadmill speed from the GXT was also 13.5 km h^−1^ with VO2 peak 53 mL kg^−1^ min^−1^

To determine the validity of using RPE reported during a submaximal 20mSRT to predict VO_2peak_, the relationship between RPE and its corresponding speed after 8 km/h were linearly regressed to obtain the constant and *b*-coefficient and extrapolated to RPE9 and 10 to predict peak speed using Microsoft Excel (Microsoft Corp, Redmond, USA). This method has been used in numerous studies (Coquart et al. 2014). An example of this method is shown in Fig. 1. RPE9 and 10 were utilised as children typically report terminal RPE values that are lower than the theoretical maximum following termination of an exercise test (Lambrick et al. 2011). Predicted peak speed was then used to estimate VO_2peak_ using ACSM’s metabolic equation (ACSM 2007). The measured VO_2peak_ from the GXT, VO_2peak_ predictions at RPE9 and 10 from the first submaximal 20mSRT, and predicted VO_2peak_ from the maximal 20mSRT were analysed via a two-factor repeated measures ANOVA [Gender(2)*Method(4)] using SPSS.

#### Reliability analysis

To examine the reliability of the predicted peak speed at RPE9 and 10 between the two submaximal 20mSRTs, a two-way mixed effects intraclass correlation coefficient (ICC) based on single measurement were calculated using SPSS.

#### Affective responses analysis

To assess affective responses between tests, a three-factor mixed-model ANOVA [Gender (2) * Time (2)*Test (4)], with repeated measures on time and test was conducted.

Significance level of 0.05 was set for all statistical tests. Where sphericity was violated, the Greenhouse–Geisser correction was applied. Repeated measures planned comparisons were used to identify the nature of main effects or interactions. Partial eta^2^ (*η*_*p*_^2^) were reported for all ANOVA.

## Results

Of the 32 enrolled participants, 2 dropped out and 5 were excluded due to not completing the protocol as intended (e.g., stopping prior to reaching RPE7 during the submaximal 20mSRT). Therefore, 25 children (*n* = 14 boys) were included in the analysis. An independent samples t-test was conducted to determine any significant differences in descriptive characteristics between gender (Table 1). VO_2peak_ (*t*_23_ = 3.325, *p* < 0.01) and VT (*t*_23_ = 3.157 *p* < 0.01) were significantly higher in boys. No other significant differences were observed.

**Table 1:** Descriptive characteristics of participants (mean ± SD)

Variable	All (*n* = 25)	Boys (*n* = 14)	Girls (*n* = 11)
Age (years)	12.8 ± 0.7	12.6 ± 0.6	12.9 ± 0.7
Height (cm)	162.0 ± 9.3	160.1 ± 11.2	164.4 ± 6.0
Mass (kg)	49.9 ± 7.7	48.0 ± 8.1	52.3 ± 6.6
Body Mass Index (BMI; kg m^−2^)	18.9 ± 1.8	18.6 ± 1.8	19.3 ± 1.8
VO_2peak_ (mL kg^−1^ min^−1^)*	46.0 ± 6.3	49.1 ± 5.2	42.0 ± 5.5
HR_max_ (beats.min^−1^)	192.9 ± 8.0	192.9 ± 7.9	192.9 ± 8.4
RER	1.08 ± 0.06	1.07 ± 0.06	1.09 ± 0.07
VT (mL kg^−1^ min^−1^)*	29.8 ± 6.2	32.8 ± 5.5	26.1 ± 4.9
VT as % of VO_2peak_ (%)	64.4 ± 7.0	66.6 ± 7.6	61.8 ± 5.3
RPE at VT (GXT)	3.6 ± 0.6	3.4 ± 0.6	3.8 ± 0.6
Terminal RPE (GXT)	9.6 ± 0.5	9.5 ± 0.5	9.7 ± 0.5
Terminal RPE (max.20mSRT)	9.8 ± 0.4	9.7 ± 0.5	9.9 ± 0.3
Peak GXT speed (km h^−1^)	11.9 ± 1.5	12.1 ± 1.4	11.5 ± 1.5
Peak 20mSRT speed (km h^−1^)	11.6 ± 1.4	12.1 ± 1.4	11.1 ± 1.1

VO_2peak_, peak oxygen uptake; HR_max_, maximal heart rate; RER, respiratory exchange ratio; VT, ventilatory threshold; RPE, ratings of perceived exertion; GXT, graded exercise test; 20mSRT, 20-m Shuttle-run test

*Significant gender difference (*p* < 0.01)

### Strength of individual linear regression model of RPE:speed

The *R*^2^ values provided strong justification for the choice of a linear extrapolation model for the line of best fit for each individual RPE:speed relationship. For the first and second 20mSRT, the average *R*^2^ values were 0.95 (SD 0.04, range 0.83–1.00) and 0.96 (SD 0.02, range 0.92–1.00).

### Validity of prediction equations to estimate VO_2peak_

A comparison of the predicted mean VO_2peak_ based on the maximal 20mSRT performance using all equations are shown in Table 2. The ANOVA revealed a significant main effect for method (*F*_2.410, 57.836_ = 78.783, *p* < 0.001, *η*_*p*_^2^ = 0.766). Post-hoc pairwise comparisons revealed all equations used to estimate VO_2peak_ were significantly different to measured VO_2peak_. The ACSM equation provided the closest estimate (mean difference = − 3.7 mL kg^−1^ min^−1^, *p* < 0.001) and was used for subsequent analyses.

**Table 2:** Comparison of mean estimated VO_2peak_ from various prediction equations compared to the GXT

Equation	VO_2peak_ (mL kg^−1^ min^−1^)	Mean difference	Significance (*p*)
ACSM	42.3 ± 4.6	− 3.7	< 0.001
Barnett	51.4 ± 6.1	5.4	< 0.001
Leger	50.1 ± 7.2	4.1	< 0.001
Mahar	55.1 ± 7.9	9.1	< 0.001
Matsuzaka	51.4 ± 6.8	5.4	< 0.001

*α* = 0.05. Mean diff. = mean difference between measured VO_2peak_ from GXT and estimated VO_2peak_ based on the maximal 20mSRT scores

### Prediction of VO_2peak_ from submaximal 20mSRT RPE

A comparison of measured mean VO_2peak_, predicted VO_2peak_ using the ACSM equation based on the speed–RPE relationship from the first submaximal 20mSRT that were extrapolated to RPE 9 and 10, and the maximal 20mSRT are shown in Table 3.

**Table 3:** Comparison of measured VO_2peak_ and estimated VO_2peak_ at RPE 9, 10 and maximal 20mSRT using the ACSM metabolic equation (mean ± SD)

	VO_2peak_ (mL kg^−1^ min^−1^)		
	All (*n* = 25)	Boys (*n* = 14)	Girls (*n* = 11)
GXT	46.0 ± 6.3	49.1 ± 5.2	42.0 ± 5.5
RPE_9_	44.1 ± 6.3	45.9 ± 6.8	41.9 ± 5.1
RPE_10_	46.3 ± 7.1	48.2 ± 7.6	43.8 ± 5.8
max.20mSRT	42.3 ± 4.6*	43.7 ± 4.8*	40.5 ± 3.7*

GXT, graded exercise test; VO_2peak_, peak oxygen uptake; RPE, ratings of perceived exertion; max.20mSRT, maximal 20-m shuttle-run test.

*Significant difference (*p* < 0.01) compared to VO_2peak_ measured in GXT and predicted at RPE9 and RPE10

The two factor (Gender by Method) repeated measures ANOVA revealed significant method (*F*_1.521, 34.985_ = 8.723, *p* = 0.002, *η*_*p*_^2^ = 0.275) and gender (*F*_1, 23_ = 5.186, *p* = 0.032, *η*_*p*_^2^ = 0.184) main effects. However, the gender by method interaction was not significant. Post-hoc analyses revealed significant differences between measured and predicted VO_2peak_ from the maximal 20mSRT using the ACSM equation. Importantly, however, no significant differences were observed between measured and predicted VO_2peak_ at RPE9 and RPE10.

### Reliability analysis

The ICC for predicted peak speed between the two submaximal 20mSRTs were 0.916 and 0.907 (*p* > 0.001) at RPE9 and 10, respectively.

### Affective responses analysis

**Fig. 2:**
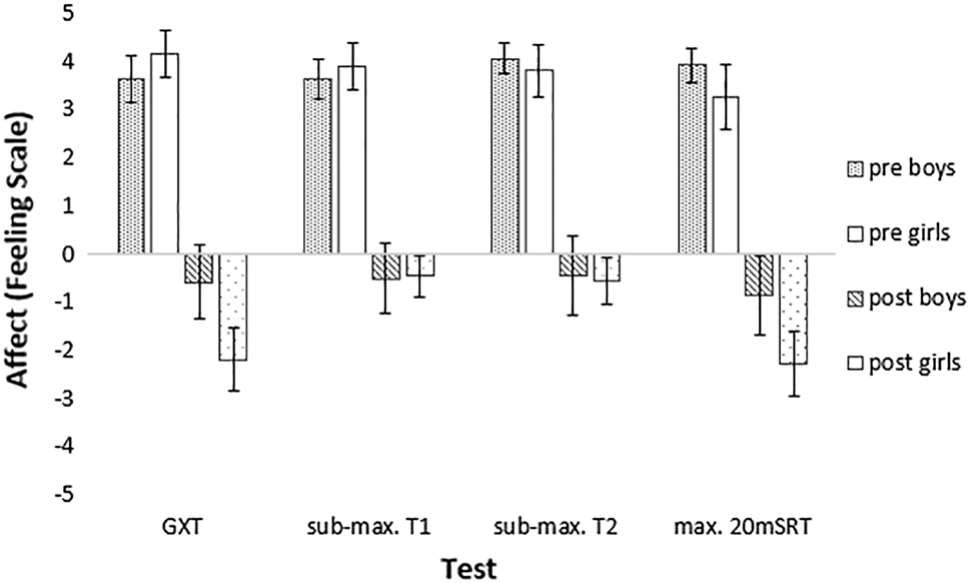
Affective responses (mean ± SE) for boys and girls, pre and end-test for all tests. *GXT* graded exercise test, *sub-max.T1* submaximal 20-m Shuttle-run test (1st trial), *sub-max.T2* submaximal 20-m Shuttle-run test (2nd trial), *max.20mSRT* maximal 20-m Shuttle-run test

The three factor (gender * time * test) ANOVA, with repeated measures on Time and Test, resulted in a significant three factor interaction (*F*_2.097, 48.237_ = 3.473, *p* < 0.05, *η*_*p*_^2^ = 0.131); significant time by test interaction (*F*_2.097, 48.237_ = 3.704, *p* < 0.05, *η*_*p*_^2^ = 0.139); and Time (*F*_1, 23_ = 90.272, *p* < 0.001, *η*_*p*_^2^ = 0.797); and test (*F*_2.071, 47.626_ = 4.506, *p* < 0.05, *η*_*p*_^2^ = 0.164) main effects. Post-hoc analyses for the time by test interaction indicated that affect was more positive pre-test, with no differences between the maximal and submaximal tests. However, end-test affect was significantly lower for all tests, with differences between the maximal and submaximal tests (Table 4). The three-factor interaction was due to end-test affective responses being significantly lower in girls for the maximal tests, relative to the submaximal tests, but with no differences between the boy’s end-test responses (Fig. 2).

**Table 4:** Comparison of mean pre-test and post-test affective response across four trials (mean ± SD)

	Pre-test	End-test
GXT	3.9 ± 1.7	− 1.3 ± 2.7^a,b^
Sub-max. 20mSRT (T1)	3.8 ± 1.5	− 0.5 ± 2.2^a^
Sub-max. 20mSRT (T2)	4.0 ± 1.5	− 0.5 ± 2.5^a^
Maximal mod. 20mSRT	3.6 ± 1.8	− 1.5 ± 2.8^a,b^

GXT, graded exercise test; Sub-max. 20mSRT, Submaximal 20-m Shuttle-run test; Max.20mSRT, Maximal 20-m Shuttle-run test

^a^Affect significantly lower than pre-test scores (*p* < 0.001)

^b^Affect lower in this test than end-test submaximal tests (*p* < 0.05)

## Discussion

The purpose of this study was to determine the validity and reliability of predicting VO_2peak_ using RPE from a submaximal 20mSRT and examine the affective responses. To our knowledge, this is the first study to use RPE in a field-based test to predict VO_2peak_ in children and to examine their affective responses. In accordance with the literature, boys had a higher VO_2peak_ compared to girls (Armstrong and Fawkner 2007).

Comparisons between measured and predicted VO_2peak_ found that equations that are commonly used in the 20mSRT overestimated VO_2peak_ in this sample of healthy 12–14-year-old children. Although predictions of VO_2peak_ from the ACSM equation were statistically lower by ~ 1 MET, the ACSM equation provided the closest prediction of VO_2peak_ when using speeds extrapolated from RPE9 and RPE10. In this study, extrapolation to RPE10 (mean difference =  ~ − 0.5 mL kg^−1^ min^−1^) provided the most accurate predictions compared to extrapolations to RPE9 (mean difference =  ~ 1.7 mL kg^−1^ min^−1^). Interestingly, our findings showed differences between measured and predicted VO_2peak_ when applying the ACSM equation to the maximal 20mSRT. The results of this study may suggest a submaximal 20mSRT protocol may provide a better estimate of VO_2peak_ in comparison to the maximal 20mSRT.

The results of this study indicate that predicting peak speed via extrapolation of RPE reported during the submaximal 20mSRT is highly reliable. A previous systematic review (Artero et al. 2011) reported ICC values ranging from 0.78 to 0.93 for the 20mSRT in youth aged 8–18 years—however, the ICCs were reported for the maximal 20mSRT. The present study, therefore, shows that despite adopting a submaximal protocol, reliability was not compromised.

Affective responses during exercise are important as they are a strong predictor of future exercise behaviours (Schneider et al. 2009). The results of this study showed no difference in pre-test affect across trials in both genders. However, significant gender differences in end-test affective responses were observed. We speculate the observed difference in end-test affective responses may potentially be based on differences in the tolerance of physical discomfort and/or pain. In experimental pain studies, women consistently report lower pain thresholds and tolerance (Bartley and Fillingim 2016). Interestingly, in boys, there were no differences in end-point affect across all trials. In girls, end-point affective responses during the submaximal 20mSRTs were in the negative domain, although these were more positive in comparison to the GXT and maximal 20mSRT. Specifically, affective responses were on average 1.7 units ‘less negative’ when girls completed the submaximal trials compared with the GXT. A difference of this magnitude is still clinically significant. As previously discussed, Schneider et al. (2009), after controlling for fitness and gender, found a one-unit increase in acute affective responses to exercise in adolescents predicted ~ 30 min of additional MVPA per week. As physical activity drops significantly during adolescence, especially in girls (Butt et al. 2011), it is critical for fitness testing methods to consider acute affective responses to support future activity behaviours.

In this study, participants were directed to stop at RPE7 in the submaximal 20mSRT. At the end of the first submaximal trial, 44% of individuals (*n* = 11) reported negative affect; and 52% (*n* = 13) reported negative affect at the end of the second submaximal trial. In this study, RPE7 on the E–P Scale corresponded to approximately 90% VO_2peak_ which is in line with previous research (Lambrick et al. 2016). Therefore, it is not surprising for the children in this study to report an overall negative end-point affect following exercise at RPE7 on the E–P Scale. Further investigation is warranted to determine whether stopping the submaximal 20mSRT earlier (e.g., at RPE5) could maintain positive end-test affect while providing accurate predictions of VO_2peak_. If found to be valid, this method could provide an alternative to field-based fitness testing while promoting positive exercise experiences in youth which may be important for future PA behaviours. In this regard, in the treadmill study of Lambrick et al. (2016), it is worth noting that predictions of VO_2peak_ extrapolated from RPE5 (80% VO_2peak_) on the E–P Scale, while not statistically different, were less accurate compared to extrapolations from RPE7.

This study has limitations that should be addressed. The 20mSRT is commonly conducted in groups. However, adopting a pragmatic approach, in this study, participants performed all trials by themselves and, therefore, may lack ecological validity. As peer/group influence may play a role in mediating RPE response (Haile et al. 2015), further investigation is needed to determine the validity of using RPE during a submaximal 20mSRT to predict VO_2peak_ in a group setting.

This study was also limited to healthy-weight population, and therefore, the results may not be translatable to children/adolescents who are underweight, overweight or obese. In the current study, the cardiorespiratory fitness of the sample was within typical range. However, due to convenience sampling and the nature of the study, bias may be present as children who are more ‘sports-oriented’ may likely have opted to participate. In addition, the pubertal status was not measured.

## Conclusions

The results of this study provide evidence for the utility of RPE reported during a submaximal 20mSRT protocol to predict VO_2peak_ accurately and reliably. The use of a submaximal 20mSRT protocol ending at RPE7 may provide better predictions of VO_2peak_ while minimising aversive end-point affective responses, especially in girls.

Acute affective responses to exercise are important to consider as they are a strong predictor of future PA behaviour. This study is the first to utilise RPE reported during a submaximal 20mSRT to predict VO_2peak_ in children and measure affective responses. Measured VO_2peak_ from a GXT using direct online gas analysis was used as a criterion to determine the most accurate prediction equation for the 20mSRT. Furthermore, a test-re-test design was utilised to determine the reliability of the submaximal 20mSRT. The findings from this study may help to inform future research and the current practice of the 20mSRT as a fitness testing battery.
